# Methods of Biotyping of* Streptococcus mutans* Species with the Routine Test as a Prognostic Value in Early Childhood Caries

**DOI:** 10.1155/2017/6859543

**Published:** 2017-06-15

**Authors:** Wirginia Krzyściak, Dorota Kościelniak, Monika Papież, Anna Jurczak, Palina Vyhouskaya

**Affiliations:** ^1^Department of Medical Diagnostics, Faculty of Pharmacy, Jagiellonian University Medical College, Medyczna 9, 30-688 Kraków, Poland; ^2^Department of Pediatric Dentistry, Institute of Dentistry, Jagiellonian University Medical College, Kraków, Poland; ^3^Department of Cytobiology, Faculty of Pharmacy, Jagiellonian University Medical College, Kraków, Poland

## Abstract

**Purpose:**

In order to investigate the suitability of* Streptococcus mutans* species biotyping by measuring the activity of selected enzymes from a commercial test, criteria were established for biotyping clinical strains from children with dental caries. In addition, the relationships between the selected biotypes, sensitivity to commonly used antibiotics, and early childhood caries were determined.

**Methods:**

A total of 142* S. mutans* isolates from dental plaque of children with caries were divided into different biotypes. Patients were divided into two groups: noncavitated (1-2 in ICDAS) and cavitated (5-6 in ICDAS) lesions. Biotyping criteria were determined based on both the arbitrary method and the clusterization method. The susceptibility of the strains to amoxicillin, cefazolin, erythromycin, and teicoplanin was studied by diluting a solid medium.

**Results:**

Biotype I was the most common. Mean MIC values showed that the strains belonging to biotypes II and IV were the most sensitive to amoxicillin. For predetermined biotypes, observed differences were dependent on the severity of dental caries.

**Conclusions:**

The proposed method of* S. mutans* strains biotyping is relatively quick and simple to use, provided the application of suitable biotyping criteria, and may contribute to the effective prevention of dental caries induced by* S. mutans*.

## 1. Introduction

Dental caries is a chronic disease occurring widely in the human population and is the most common childhood disease. This is a multistage morbid process which, in addition to microbiological agents, also includes the susceptibility of host tissues to microbiological agents, the presence of carbohydrates in the oral cavity, and a sufficiently long duration of action of all these factors on the tooth enamel.

The microorganism* Streptococcus mutans* is considered one of the main etiological factors of dental caries. This commensal microorganism has a number of features that in appropriate environmental conditions determine its pathogenicity. Penetration of the physical and immunological protective host barriers by* S. mutans* leads to an invasion by the microorganism and consequent infection of the body. Most infections caused by* S. mutans* are a consequence of its proliferation in the human body. There are listed cases when* S. mutans* has induced endocarditis and bacteremia [1–3] or has been isolated from the heart valve of a patient with endocarditis [4]. Recurrent bacteremia in a woman with Sjögren's syndrome was also caused by this microorganism [5]. In addition to endocarditis and bacteremia,* S. mutans* may induce sepsis [6] or may be isolated from patients after ischemic stroke [7]. It is presumed that* S. mutans* can cause other systemic diseases [8]. A rare case of retroperitoneal abscess caused by* S. mutans* has also been described [9].

Adverse effects of the microorganism's activity may be reduced by using prophylaxis or by an effective immune response from the host. On the other hand, excessive host immune response may lead to self-destruction of the organism as a result of the protective role of the organism against the bacteria (own unpublished studies).

A thorough study of* S. mutans* biotypes is necessary to determine the differences in susceptibility to selected antibiotics and subsequent effective prevention of infection by this microorganism. Moreover, the correct classification of the* S. mutans* species in selected biotypes may shed new light on their exact origin and the incidence of various infections concomitant to caries, including bacteremia or ischemic stroke, making effective prevention by healthcare professionals easier to carry out.

There are various methods for typing* S. mutans* strains; one of the earliest introduced methods is agglutination-based serotyping [10] or typing based on auxonogram, which uses intraspecies differences in the ability to assimilate various organic compounds. Depending on the compounds used, there are different variations of this method. Quite popular in routine diagnostics is the typing of* S. mutans* strains upon auxonograms and enzymograms of STREPTOtest 24 (Lachema, Pliva) [11], as well as API 20 Strep (bioMérieux) [12].

Another typing method is biotyping based on selected reactions of commercial enzymatic tests. Biochemical typing may have similar usefulness in terms of the differentiation of group B streptococci as serotyping [13]. Biotyping is applicable to the streptococci of* Streptococcus mutans* group (*S. mutans* and* S. sobrinus*) isolated from dental plaque [14].

This article focuses on both the identification of the biotyping criteria for clinical* S. mutans* strains isolated from children with varying degrees of severity of dental caries and the assessment of the relationships between the selected biotypes in vitro, sensitivity to commonly used antibiotics, and severity of dental caries in children.

## 2. Materials and Methods

A total of 143* S. mutans* strains were isolated from supragingival plaque (scraped from the tooth surface using a sterile dental excavator) of the molar milk teeth of children with early caries (*n* = 143; average age: 4.6 ± 0.76 years). Criteria for the examinations were in accordance with Oral Health Surveys Basic Data (World Health Organization) [15]. Examinations were conducted with the use of a dental mirror and a probe in artificial light. The ICDAS (International Caries Detection and Assessment System) was applied to classify the severity of tooth decay and its division into study groups (1: first changes in enamel visible “only after prolonged air drying or restricted to within the confines of a pit or fissure”; 2: distinct visual enamel changes, no loss of surface integrity; 3: localized enamel breakdown; 4: underlying dentin shadows; 5: distinct cavities, visible dentin; 6: extensive distinct cavities, visible dentin) [16]. The daily oral hygiene of patients involved in the study comprised brushing teeth twice a day using a manual brush and a fluoride toothpaste. The exclusion criteria comprised age <2 or >6 years, diabetes, epithelial dysplasia, periodontal disease, inflammatory lesions of the oral mucosa, and use of antibiotics, nonsteroids, anti-inflammatory medications, corticosteroids, and vitamins within the last 3 months.

Patients were divided into two groups: a noncavitated group with initial tooth decay (ICDAS 1-2; *n* = 97, average age: 4.64 ± 0.77 years, including 47 females aged 4.68 ± 0.91 years and 50 males aged 4.6 ± 0.61 years) and a cavitated group with extensive decay (ICDAS 5-6; *n* = 46, average age: 4.52 ± 0.75 years, including 20 females aged 4.75 ± 0.85 years and 26 males aged 4.35 ± 0.63 years). The division criterion between the two groups was the lack/presence of visible carious tissue loss.

Dental plaque was evaluated with the Simplified Oral Hygiene Index (OHI-S) which assesses its presence on 6 teeth of different groups (55, 53, 51 and 75, 73, 71), lingual surfaces of the mandibular teeth, and labial surfaces of the maxillary teeth (0: lack of calculus; 1: calculus covers <1/3 of the tooth surface; 2: calculus covers 1/3–2/3 of the surface; 3: calculus covers >2/3 of the tooth surface) [17]. The OHI-S in all examined children was equal to 1.

Before the examination, patients remained fasting and did not clean their teeth on the day of the study. Material was collected after rinsing the oral cavity with distilled water in the morning (before 11 a.m.). The collected plaque was placed in Eppendorf sterile tubes with 0.5 ml of saline without access to atmosphere oxygen and transported to the laboratory on ice within 2 h. Subsequently, the samples were sonicated for 20 s at an amplitude of 5 *µ*m, vortexed to obtain a homogeneous suspension, and centrifuged at 1,000*g* at a temperature of 4°C for 15 min. A total of 100 *µ*l of the supernatant was used in conventional growth methods.

Levels of* Streptococcus mutans* in plaque were determined using selective agar. The plaque was inoculated on a HLR-S agar medium in the laboratory. It was assumed that the role of the medium consisted of support for the growth of the tested microorganisms and inhibition of the remaining species found in the saliva. The incubation time was 48 h under microaerophilic conditions (85% N_2_, 10% CO_2_, and 5% O_2_) at 37°C. The HLR-S medium was selected based on our previous study, where we had tested media described in the literature as being selective for specific bacterial species isolated directly from the oral cavity (from plaque or saliva samples) [18, 19].

**Figure 1:**
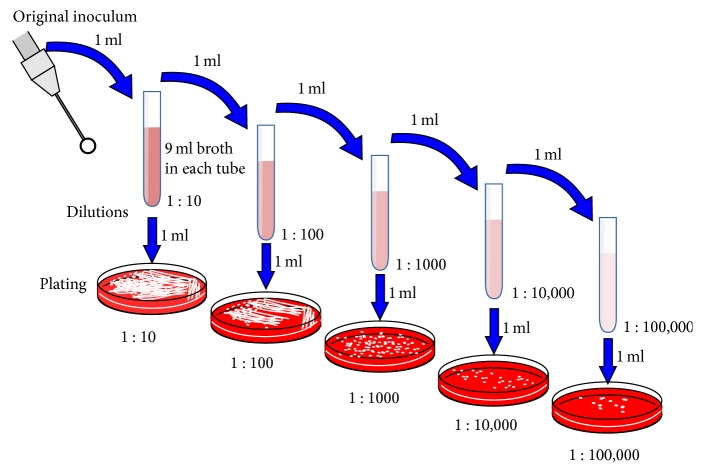
Preparations of the dilutions of bacterial suspension.

Individual cultivated colonies were inoculated on the TSA medium with 5% sheep blood and incubated for 24 h under the above conditions. Then, the morphological characteristics of individual colonies cultivated on the medium with sheep blood were evaluated, as was the type of hemolysis caused by these colonies. In addition, plaque was formed, and this was inoculated on the HLR-S medium. The colonies were counted via determination of CFU/ml (Figure 1). The number of microorganisms present in a particular test sample was determined using the formula

(1) $$\begin{array}{c} CFU/ml=CF \end{array}$$

Cultures of microorganisms were carried out under microaerophilic conditions and visualized with Gram staining under a light microscope, as well as under an electron microscope.

**Figure 2:**
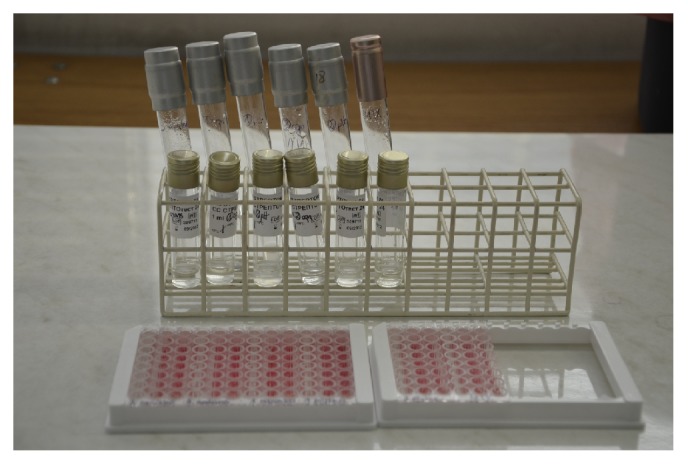
Prepared identification STREPTOtest 24 tests (Lachema, Pliva). In the stand, there are bacterial suspensions in saline, suspension media for STREPTOtest 24, and identification panels established in accordance with the manufacturer's instructions.

Phenotypic identification was performed using a commercially available assay for the streptococci identification (STREPTOtest 24, Lachema). The enzymes used in the study are given in Table 1. Isolated strains were cultured on HLR-S medium for two days under microaerophilic conditions. The single colonies of bacteria once grown were inoculated on blood agar (BA) and incubated for two days at 36°C, 5% CO_2_ conditions. A suspension of pure colonies was prepared in a 3–3.5 ml sterile NaCl solution to obtain 2–2.2 on the McFarland scale (Figure 2). A total of 100 *μ*l of this bacterial suspension was applied to each of the wells of the first row (designated as NAG, LAP, bMN, GLR, bGL, bGA, Aga, and PHS) and to wells in column H (designated as ESL and ARG). 1.5 ml of the above suspension was transferred to a NaCl solution as a medium for the STREPTOtest 24 suspension (Lachema, Pliva). A total of 100 *μ*l of the thus prepared solution was applied to each of the remaining reaction wells (INU, MAN, SOR, MLB, RIB, LAC, PUL, S06, AMG, TGT, MLT, RAF, TRE, and SOE). A drop of paraffin oil was added to the plate wells designated as Arg and S06.

**Figure 3:**
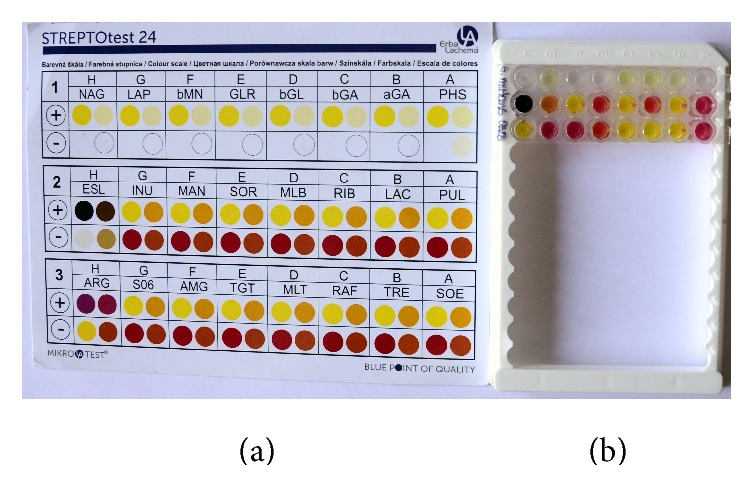
Comparative color scale (a) and STREPTOtest 24 identification test (Lachema, Pliva) after incubation (b). The test result was evaluated based on the color scale shown above.

Following the transfer of the prepared solutions, the specimens were incubated for 24 h at 37°C. The reading was based on a visual assessment with the comparative color scale supplied by the manufacturer. The results were interpreted as “positive” or “negative” and were represented on a 7-point scale (0–7) depending on the intensity of the color reaction (Figure 3). Species were identified based on the codebook provided by the manufacturer (the bacterial species are encoded).

Grouping of 143 identified* S. mutans* strains was performed based on the differences in the activity of selected enzymes from the commercial test used in diagnostics. Out of the 24 enzymes present in the STREPTOtest 24, those enzymes were selected for biotyping, whose occurrence among the tested strains was in the 15–85% quartile deviation, that is, inulin (40.56%), melibiose (83.22%), and tagatose (21.68%) (Table 2).

The assay was performed in triplicate for each strain, and each set of tests was evaluated independently by a second observer. Subsequently, 143 strains were tested for sensitivity to the selected classes of antibiotics, including the *β*-lactams (amoxicillin, cefazolin), the macrolides (erythromycin), and the glycopeptide antibiotic (teicoplanin), using a dilution method in a solid phase in the concentration range 0.007–1.5 mg/L [20]. The statistical significance of differences in susceptibility to selected antibiotics in particular biotypes was determined using the Kruskal-Wallis test complemented by post hoc analysis (Dunn's test).

In the clustering process, the following methods were used: hierarchical clustering, scree plot showing decrease in ESS (error sum of squares), and an objective method (silhouette).

## 3. Results

### 3.1. Phenotypic Identification

#### 3.1.1. Morphological Characteristics

**Figure 4:**
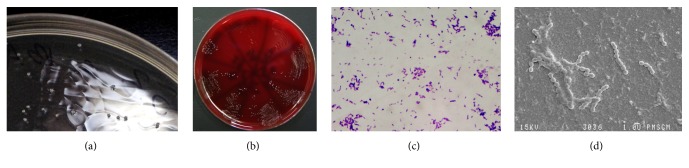
*Streptococcus* sp. cells isolated from dental plaque from a child with ECC. The picture shows the macro- and micromorphology of* Streptococcus* sp. (a)* S. mutans* colonies with a characteristic white color on the HLR-S selective medium; (b) on blood agar BA; (c) Gram-stained* S. mutans* cells (Olympus CX41, CaMedia C5550); (d)* S. mutans* cells under a scanning electron microscope.

**Figure 5:**
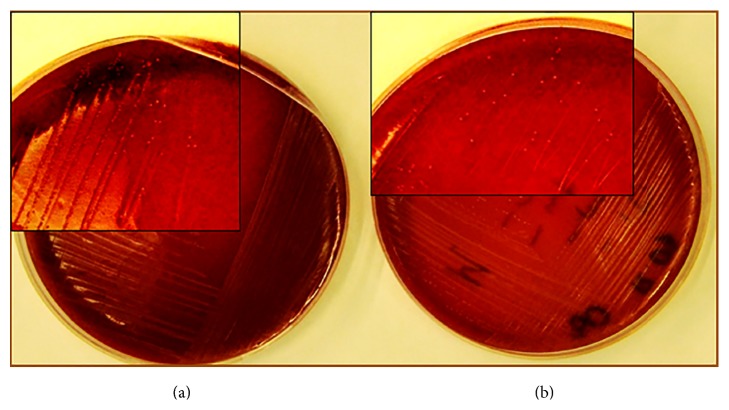
Macrophotography of the* S. mutans* species. (a)* S. mutans* standard strain ATCC 700610. (b)* S. mutans* strain isolated from the clinical material. Visible characteristic small white colonies and the absence of hemolysis on the blood substrate.

The isolated bacterial species were Gram-stained and viewed under a light microscope at an objective magnification of 40x. The size of the observed colonies ranged from 0.5 to 1 *μ*m. The spatial arrangement of cells resembled chains long as beads (Figure 4). The tested species of bacteria did not produce catalase. On a medium supplemented with sheep blood, they demonstrated *γ*-hemolysis (Figure 5).

Two approaches were used in determining biotypes among the 143 isolated* S. mutans* strains. The first approach was to select particular enzymes based on the analysis of their activity in the population of tested strains. Five enzymes present in all strains (reactions referenced as LAP, MAN, LAC, and TRE), the growth test in the presence of 6.5% NaCl, which was negative for all strains, and seven enzymes that were not present in any strain (bMN, GLR, PUL, ARG, S06, AMG, and SOE) were not suitable for differentiation and were rejected per se. Another seven enzymes occurring in these cases were also rejected, because the use of these enzymes in biotyping would have resulted in a heterogeneous distribution of strains in particular biotypes (there would have been too many biotypes containing one strain):*β*-glucuronidase occurring in 90.21% of the strains,*β*-galactosidase (90.91%), *α*-galactosidase (90.21%), sorbitol (90.91%), ribose (11.89%), phosphatase (11.88%), maltose (86.01%), and raffinose (86.01%).

When criteria for including enzymes into biotyping are narrowed to 10–90%, 7 enzymes are left giving 24 profiles, or 12 biotypes. When the range 12.5–87.5% is considered, only 5 enzymes are left giving 18 profiles and 9 biotypes. When the 15–85% range is considered, 3 enzymes are obtained, giving 8 profiles (Table 3) and finally 4 biotypes (Table 4).

We decided to choose the latter, because, given the 143 samples, even a biotype number equal to 9 would be too large (on average, this gives less than 16 samples per biotype).

Enzymes chosen for biotyping were INU, MLB, and TGT. The adoption of these enzymes as the biotyping criteria allowed the specification of 8 enzymatic profiles, conventionally defined with consecutive letters of the alphabet from A to H, as shown in the table (Table 3). Profile C characterized by a lack of activity of inulin and tagatose is the most numerous.

Resolution of the obtained profiles is heterogeneous, and therefore strains whose profiles differ only in MLB (MLB is the nearest to being excluded from the 15–85% range) were grouped into one biotype. The four such biotypes are presented in Table 4.

The second method of determining biotypes uses unsupervised (available without a priori knowledge) statistical data analysis. The data were divided into groups (clusters), so that each of the groups was as homogeneous as possible (the strains within a cluster are related to each other), while clusters were different (strains from different groups have as few common features as possible).

**Figure 6:**
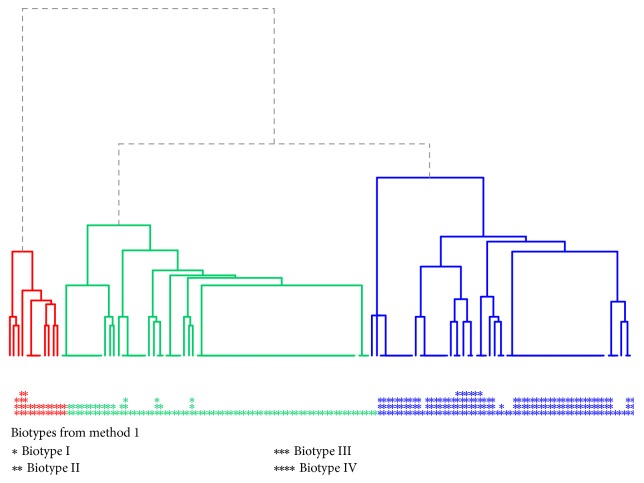
Dendrogram with 3 marked* S. mutans* biotypes in the studied population.

The results of the division into clusters are displayed as dendrograms (diagrams in the shape of a tree showing the relationships and similarities between selected elements). In the case of the most well-fitted dendrogram, strains could be differentiated from the activity of melibiose and tagatose. Thus, 3 biotypes (the main branches of the diagram, Figure 6) were obtained and are marked with the colors red, green, and blue. The figure also indicates the distribution of enzymatic profiles obtained using the arbitrary method: the green cluster, biotype I; red, biotype II; and blue, biotype III.

**Figure 7:**
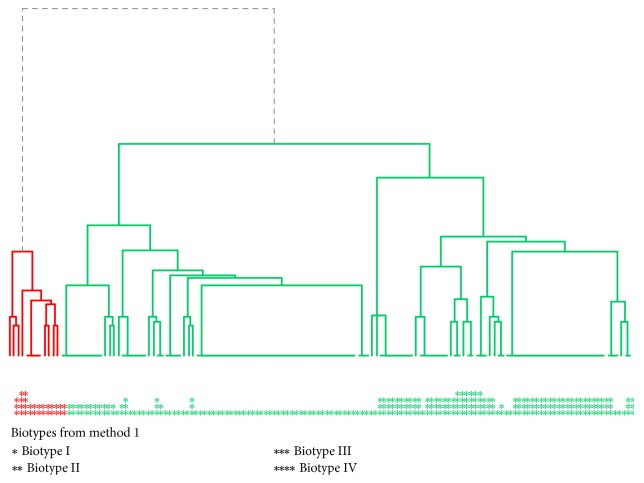
Dendrogram with 2 marked* S. mutans* biotypes in the study population determined by the objective method (silhouette).

Additionally, the number of clusters was confirmed using an objective profiling methodology (silhouette method) which gave the dendrogram presented in Figure 7. The analysis gave 2 clusters marked in the colors red and green. In this method, the number of clusters was selected using the objective method (silhouette). In our observations, the criterion for the rejection of the abovementioned method is that one cluster has 11 observations, while the second has above 100.

**Figure 8:**
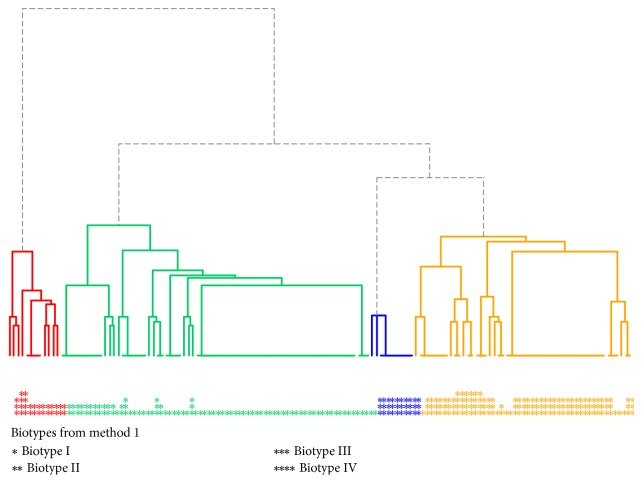
Dendrogram with 4 marked* S. mutans* biotypes in the study population determined by a subjective arbitrary-based method. Colors show the division into 4 obtained biotypes as a dendrogram; stars under it show biotypes of the arbitrary method.

Using the results obtained in the arbitrary method, a third dendrogram was created showing 4 clusters (Figure 8). The distribution of biotypes obtained in the abovementioned method is as follows: the red cluster is mainly biotype II, but also III and IV; the green cluster is mainly biotype I, but also II and III; the blue cluster is only biotype III; and the yellow one is mainly biotype III, but also I, II, and IV.

**Figure 9:**
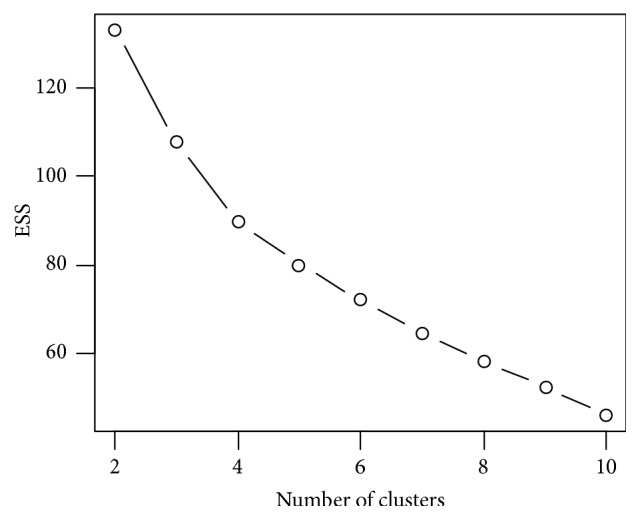
Determining the optimal number of clusters using the scree plot method.

In addition, a “scree plot” method was used in the identification of the optimal number of clusters (Figure 9). The result of the analysis appears to be consistent with the established arbitrary method, which coincides with the set dendrogram (Figure 8). The graph shows that the elbow is for 4 clusters (because ESS drops sharply up to 4 clusters and then more smoothly). Thus, this method points to 4 clusters, as determined in Figure 8.

After analyzing all three dendrograms, the clustering shown in Figures 6–8 appears reasonable, although the dendrogram in Figure 6 appears to be more consistent from the clinical point of view, because one cluster is formed by strains belonging to one biotype. This is as unlikely as in the case of the dendrogram shown in Figure 8 (one cluster is formed by several strains of different biotypes). A comparison of the biotypes obtained in the two methods is shown in Tables 5 and 6.

As can be seen in the presented tables, the division into four clusters, despite being supported by an objective method, does not quite make sense from a clinical perspective. In the case of the dendrogram with 4 clusters, there are two clusters where the classified strains constitute less than 7% and 8% of strains, respectively. In addition, the distribution of strains in clusters is heterogeneous; that is, one cluster is formed by strains of at least 3 or even 4 biotypes as a result of the arbitrary method. In the case of three clusters, the distribution of strains is relatively homogeneous and grouped within the selected specific biotypes.

### 3.2. Advanced Form of Dental Caries in Certain Biotypes

#### 3.2.1. Arbitrary Method

**Figure 10:**
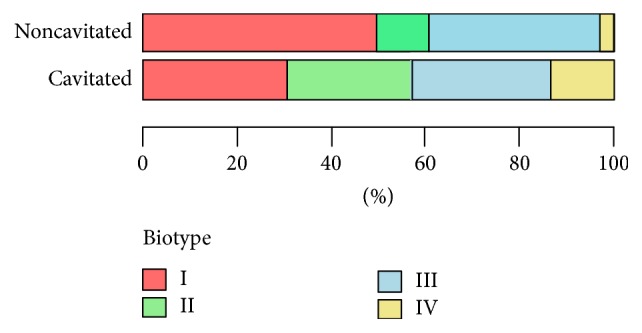
The analysis of the obtained biotypes (arbitrary method) in terms of the severity of dental caries. Fisher's exact test; *p* < 0.05.

While analyzing the 4 biotypes obtained using the arbitrary method in terms of the severity of caries, it was noted that the analyzed groups differed significantly (*p* < 0.05; Fisher's exact test) in the occurring biotypes (Table 7, Figure 10).

The analysis shows that biotypes obtained in the arbitrary method do not have a specific use in patients with varying degrees of dental caries severity. The number of strains belonging to biotype IV is too low: 2 strains (2.06%) for the noncavitated group and 6 strains (13.04%) for the cavitated group. Therefore, these criteria were rejected during the assessment of the relationship between the selected biotypes and caries severity (Table 7, Figure 10).

#### 3.2.2. Clusterization Method

**Figure 11:**
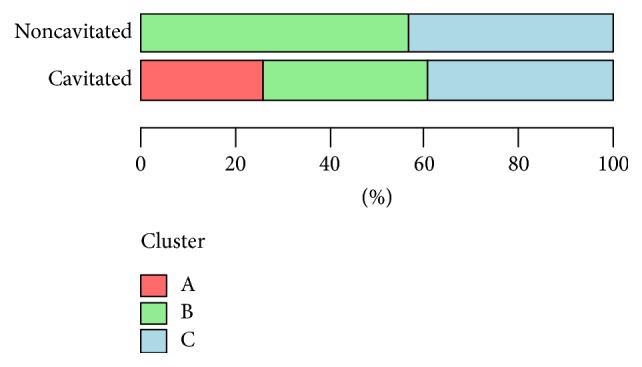
Analysis of the obtained clusters (clusterization method) in terms of the severity of dental caries. Fisher's exact test; *p* < 0.05.

While analyzing biotypes in the most well-fitted dendrogram in terms of the severity of caries, it was noted that the analyzed groups differed significantly (*p* < 0.05; Fisher's exact test) in the occurring clusters (Table 8, Figure 11).

Although no strain occurred in the noncavitated group in cluster A, the present biotype arrangement seems more reasonable from the clinical point of view. The red cluster is formed by strains from biotype II alone, which lack the activity of inulin, but with active tagatose and partially active melibiose; the green cluster is formed by strains from biotype I lacking the activity of inulin and tagatose, with partially active melibiose; the blue cluster is strains of biotype III, with inulin activity and partially active melibiose and without active tagatose. In addition, the distribution of strains in clusters is comparable (Table 8, Figure 11).

### 3.3. Sensitivity to Antibiotics in Certain Biotypes

A comparison of mean MIC values for each antibiotic was undertaken, and the range of MIC and MIC_50_ values is given in Table 9. It has been demonstrated that strains from certain biotypes show different levels of susceptibility to selected antibiotics. As is apparent from the mean MIC, strains belonging to biotype IV were more sensitive to amoxicillin and cefazolin (*p* < 0.01) than to teicoplanin (*p* < 0.001) and erythromycin (*p* < 0.001). Among the strains isolated from children with caries (cavitated group), the strains of biotypes II and IV were the most sensitive to amoxicillin (*p* < 0.01). In the case of strains belonging to biotype IV, strains from the noncavitated group were the most sensitive to amoxicillin (*p* < 0.01). Results obtained in the arbitrary method concur with the results from the clustering into 3 clusters, wherein strains belonging to cluster A (mainly biotypes II and IV) were the most susceptible to amoxicillin (*p* < 0.001) (Table 10). Among the strains isolated from children with caries (cavitated group), strains belonging to cluster A (biotype II, arbitrary method) were the most sensitive to amoxicillin (*p* < 0.001) and erythromycin (*p* < 0.001). In the noncavitated group, strains of cluster C (biotypes III and IV) were the most susceptible to amoxicillin (*p* < 0.01). According to the obtained mean MIC values, amoxicillin was the most effective in biotypes II and IV (similarly in cluster A), and erythromycin was the most effective in biotypes I and II (Table 9). The factor supporting the comparative effectiveness of this method is its reproducibility in the two methods (arbitrary and clusterization method, available without a priori knowledge). Due to the relatively quick and simple procedure, this method may be a valuable prognostic tool in studies of* S. mutans*, especially in terms of the prevention of dental caries in children.

## 4. Discussion

Via evolution, organisms adapt to the environment which they inhabit. Features they developed facilitate their search for food, survival, and proliferation. An extreme example of adaptation to the environmental conditions is obligate parasites, for example,* Plasmodium falciparum*, which need a definite host to finish their life cycle. The majority of human pathogenic bacteria occur naturally, mostly in the soil, water, or decaying organic matter, and do not require hosts for propagation. The broad adaptability of pathogenic bacteria allows them to be both saprophytes and parasites, depending on the location and conditions under which they currently exist. One should keep in mind that even pathogenic bacteria are opportunists and, as discussed in the Introduction, the occurrence of bacteremia requires many factors. Not only the properties of a bacterial cell but also the conditions in which it exists are important, in this case the host immune condition. Some bacteria, such as facultative anaerobes, seem to prefer a special environment: the human oral cavity [21].

Approximately five quintillion (5 × 10^30^) bacteria have been identified, and it is estimated that there may be about 5 × 10^60^ in total [22]. Only a few hundred of these species have been described in cases of human infections [23]. Pathogenic bacteria have developed a number of features that enable them to survive and reproduce in a living organism. It can be assumed that some more common pathogens are more adapted to a parasitic lifestyle than others, which may be at the beginning of the “road” to parasitism. Questions arise as to which features distinguish pathogenic species from species unable to infect, which features are essential for a bacterium to parasite in mammals, and what differs commonly isolated pathogens such as* S. pyogenes* from newly emerging pathogens such as* S. mutans*.

Awareness of the characteristics determining the pathogenicity of newly appearing species responsible for bacteremia can contribute to an understanding of the general mechanisms of bacterial infections. Comparison with documented bacterial pathogens will determine which features are most important in the evaluation of bacterial pathogenicity.

The aim of this study was to determine the relationship between the established biotypes among* S. mutans* strains isolated from children with caries, caries severity, and sensitivity to selected antibiotics.

In our study, we focused our attention on* S. mutans* strains isolated from dental plaque, a recognized etiological factor of caries. We selected isolates of bacterial strains based on the presence of* S. mutans* in the test material. The frequency of* S. mutans* isolation was approximately 80% in patients with early childhood caries and 24% in children without symptoms. The obtained data are consistent with our previous results [24] and also with available literature, where the frequency of* S. mutans* isolation is very diverse and the range of results is from 59% [25] through 66% [26] to up to even 100% [27].

Biotyping allows the combination of strains with similar characteristics into groups called biotypes. There are different approaches to strain typing. In the present study, we applied biotyping of* S. mutans* strains upon the profiles of enzymatic activity in a commercial assay: STREPTOtest 24.

In the case of oral streptococcosis, biotype I or the green cluster (B) dominates (*S. mutans* strains with inactive inulin and tagatose and with partial activity of melibiose). Other biotypes were observed less frequently or not at all. Interestingly, in the case of systematic infections, the dominance of biotype I is not so much in favor of other more often isolated biotypes [28]. However, it is difficult to discuss the biotypes specific to different types of infections, given the limited number of undertaken research studies.

There are no specific (conventionally adopted) biotypes and thus enzymes to classify (perform biotyping) for the* S. mutans* species. In this paper, we attempted to determine biotypes for dental caries. Two approaches to the determining of enzymes useful to biotyping were adopted. One of these was the arbitrary method of enzymes selection, where the incidence of the enzyme activity in the studied population was adopted as a criterion. On this basis, we noticed that, to differentiate strains, inulin (INU), melibiose (MLB), and tagatose (TGT) have the best activity. These results are only partly consistent with the results of Yoo et al. [14], where, similar to our study, strains with inactive inulin (INU) formed the most dominant biotype I. In contrast to our strains of biotype I which showed no tagatose activity, strains of biotype I determined by Yoo showed variable activity of tagatose and the presence of an enzyme that degraded melibiose (MLB), while our strains showed variable levels of activity for this enzyme.

The method seems to be promising, especially in the context of the current understanding of the mechanisms of vertical and horizontal bacterial transmission. Future studies should examine whether our proposed biotypes are characteristic for family members (mother, child, and sibling). This would allow the development of caries prevention programs based on screening tests in a population of healthy people or people from risk groups.

Our results on* S. mutans* susceptibility to the selected antibiotics are opposite to the results of Longman et al. [29] and Bryskier [30], whose amoxicillin-resistant strains showed sensitivity to erythromycin. In our case, the strains of biotypes III and IV, which were less sensitive to erythromycin, were found to be sensitive to amoxicillin. These results are consistent between the two used biotyping methods (arbitrary division into biotypes and the method of clusterization into three clusters). In the case of dental patients in the United Kingdom who received amoxicillin as prophylaxis, most isolates were resistant to erythromycin. The reason for the occurrence of erythromycin-resistant strains, especially in patients taking amoxicillin prophylaxis, is not known. Our results are similar to the results of Harrison et al. [31], who found no strains resistant to erythromycin among oral streptococci from healthy volunteers. However, it is difficult to compare the results obtained in this study with previous reports due to differences in methodology, target group, and criteria for determining the resistance to selected antibiotics. In the oral streptococci, resistance to amoxicillin and erythromycin has been studied with variable feature [32]. It can be assumed that the variable sensitivity to antibiotics of oral streptococci is dependent on the changing maturity of the mouth microbiome. This happens in the case of* Streptococcus salivarius* appearing during adolescence of oral flora in the individual life. This highlights the potential role of this species in the shaping of sensitivity of the mouth and throat to antibiotics [33, 34].

In the case of viridans group streptococci (VGS) which most often occur in the oral cavity, opportunistic species (emerging pathogens) may also occur, and these are responsible for such rare diseases as infectious endocarditis (IE), bacteremia, or ischemic stroke [35, 36]. In these cases, it appears to be decisive to determine the antimicrobial susceptibility of VGS which belong to the oral microflora and may be a potential risk factor for the development of the abovementioned diseases in dental patients.

In our identification of the bacteria isolated from the oral cavity belonging to the VGS using an API Strep test (bioMérieux, France), we determined the susceptibility of strains (MIC) to penicillin, ampicillin, and vancomycin. The most frequently isolated species of oral cavity microbiota include, for example,* S. mutans*, and 13% of these species are resistant to penicillin, while all are sensitive to vancomycin. It follows that antimicrobial resistance of VGS should be measured routinely and regularly in the local community. Based on the results of bacterial susceptibility to certain antibiotics and the forming of drug resistance among the strains of the specific biotypes, it should be possible to take preventive and therapeutic decisions in risk groups, including pediatric patients predisposed to the development of diseases including caries. In addition, one should consider empirical treatment of patients from the so-called “infective window” and groups at risk of acute VGS infections [37].

**Figure 12:**
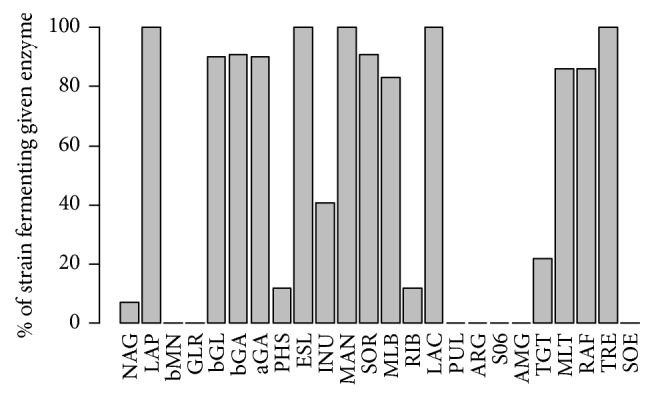
Enzymatic activity in STREPTOtest 24 of bacteria from* S. mutans* species.

In our study, a varying degree of sensitivity by* S. mutans* strains can be observed in the selected biotypes depending on the severity of dental caries. Strains grouped in clusters A and B from children with advanced caries (cavitated group) showed sensitivity to amoxicillin and erythromycin (*p* < 0.001). Strains grouped within clusters B and C from the noncavitated group showed similar sensitivity to amoxicillin and erythromycin. Interestingly, the results of the analysis of sensitivity to the abovementioned antibiotics in these clusters are characterized by the determined phenotype of the tested strains; that is, strains grouped in clusters B and C at the same time were characterized by inactive tagatose and showed varying activity of melibiose. Strains forming cluster A (mainly biotype II) were characterized by a peak sensitivity to amoxicillin and erythromycin. This group includes strains of a different phenotype compared to the above-described clusters B and C, with tagatose activity but with no inulin activity and a variable melibiose activity. In all cases, a heterogeneous distribution of strains degrading/not degrading melibiose can be observed. Melibiose decay is widely discussed with regard to differentiating* S. mutans* strains [14]. Facklam described biotypes for clinical isolates of* S. mutans* from both dental plaque and blood. In that research, the frequency of the particular enzymes was comparable to those achieved in our study (above 90%, mannose, trehalose, sorbitol, and esculin) (Figure 12). In the case of melibiose, 83.22% of our strains demonstrated the activity of that enzyme, while Facklam observed this feature in less than half of the strains isolated from plaque (43%) and 88% of the strains isolated from blood [28].

Colby et al. found that strains of* S. mutans* not fermenting melibiose constitute a genetically heterogeneous group. According to these authors, the lack of distribution of this saccharide may be a result of different genetic changes, most often caused by a chromosomal deletion covering the multiple sugar metabolism operon (msm) [38]. In our case, there are too few strains fermenting melibiose to permit discussion of grouping dependent only on this feature. In addition, the heterogeneity of MLB distribution in established biotypes/clusters prevents the drawing of conclusions for the study population.

Primary care physicians should make conscious use of antibiotic therapy, especially in infections of the upper respiratory tract. Awareness of the increasing resistance of VGS bacteria to commonly used antibiotics [39, 40] should enhance preventive measures using knowledge about the available diagnostic methods allowing the determination of the prevalence of local resistance which may influence further decisions on the use of empirical therapy and prophylaxis in certain populations and also in hospital wards where the number of multidrug-resistant strains is increasing [41].

## Figures and Tables

**Table 1:** Enzymatic reaction panel contained in the STREPTOtest 24 (Lachema, Pliva).

Code	Tested enzyme	Reaction
NAG	N-Acetyl-glucosaminidase	Hydrolysis
LAP	L-Leucine-aminopeptidase	Hydrolysis
bMN	*β*-Mannosidase	Hydrolysis
GLR	*β*-Glucuronidase	Hydrolysis
bGL	*β*-Glucosidase	Hydrolysis
bGA	*β*-Galactosidase	Hydrolysis
Aga	*α*-Galactosidase	Hydrolysis
PHS	Phosphatase	Compound hydrolysis
ESL	Esculin	Compound hydrolysis
INU	Inulin	Sugar fermentation
MAN	Mannitol	Compound fermentation
SOR	Sorbitol	Compound fermentation
MLB	Melibiose	Sugar fermentation
RIB	Ribose	Sugar fermentation
LAC	Lactose	Sugar fermentation
PUL	Pullulan	Sugar fermentation
ARG	Arginine	Compound hydrolysis
S06	Growth in 6.5% NaCl	
AMG	*α*-Methylglucosidase	Hydrolysis
TGT	Tagatose	Sugar fermentation
MLT	Maltose	Sugar fermentation
RAF	Raffinose	Sugar fermentation
TRE	Trehalose	Sugar fermentation
SOE	Sorbose	Sugar fermentation

**Table 2:** The prevalence of particular enzymes.

Enzyme	Prevalence
NAG	6.99%
LAP	100.00%
bMN	0.00%
GLR	0.00%
bGL	90.21%
bGA	90.91%
aGA	90.21%
PHS	11.89%
ESL	100.00%
INU	40.56%
MAN	100.00%
SOR	90.91%
MLB	83.22%
RIB	11.89%
LAC	100.00%
PUL	0.00%
ARG	0.00%
S06	0.00%
AMG	0.00%
TGT	21.68%
MLT	86.01%
RAF	86.01%
TRE	100.00%
SOE	0.00%

**Table 3:** Enzymatic profiles of *S. mutans* obtained based on the prevalence of activity of three enzymes: INU, MLB, and TGT in the STREPTOtest 24.

Enzymatic profile	INU	MLB	TGT	*n*	Percentage
A	−	−	−	1	0.70%
B	−	−	+	10	6.99%
C	−	+	−	61	42.66%
D	−	+	+	13	9.09%
E	+	−	−	11	7.69%
F	+	−	+	2	1.40%
G	+	+	−	39	27.27%
H	+	+	+	6	4.20%

**Table 4:** Four *S. mutans* biotypes obtained based on differences in the activity of inulin, melibiose, and tagatose in the STREPTOtest 24.

Biotype	Enzymatic profile	*n*	Percentage
I	A + C	62	43.36%
II	B + D	23	16.08%
III	E + G	50	34.97%
IV	F + H	8	5.59%

**Table 5:** Comparison of biotypes of both arbitrary and clusterization (using split into 4 clusters) methods.

Biotype from the arbitrary method	Biotype from a dendrogram							
	Red		Green		Blue		Yellow	
	*n*	%	*n*	%	*n*	%	*n*	%
I	0	0.00%	55	77.46%	0	0.00%	7	14.00%
II	9	75.00%	13	18.31%	0	0.00%	1	2.00%
III	1	8.33%	3	4.23%	10	100.00%	36	72.00%
IV	2	16.67%	0	0.00%	0	0.00%	6	12.00%

**Table 6:** Comparison of the results from the arbitrary and clusterization methods (dendrogram with a division into 3 clusters).

Biotype from the arbitrary method	Biotype from the dendrogram					
	“Red”		“Green”		“Blue”	
	*n*	%	*n*	%	*n*	%
I	0	0.00%	55	77.46%	7	11.67%
II	9	75.00%	13	18.31%	1	1.67%
III	1	8.33%	3	4.23%	46	76.67%
IV	2	16.67%	0	0.00%	6	10.00%

**Table 7:** Analysis of the obtained biotypes (arbitrary method) in terms of the severity of dental caries. Fisher's exact test; *p* < 0.05. ^*∗*^Fisher's exact test (low expected values in the table).

Biotype	Noncavitated		Cavitated		*p* ^*∗*^
	*n*	%	*n*	%	
I	48	49.48%	14	30.43%	*p* = 0.003
II	11	11.34%	12	26.09%	
III	36	37.11%	14	30.43%	
IV	2	2.06%	6	13.04%	

**Table 8:** Analysis of the obtained clusters (clusterization method) in terms of the severity of dental caries. Fisher's exact test; *p* < 0.05. ^*∗*^Fisher's exact test (low expected values in the table).

Cluster	Noncavitated		Cavitated		*p* ^*∗*^
	*n*	%	*n*	%	
A	0	0.00%	12	26.09%	*p* < 0.001
B	55	56.70%	16	34.78%	
C	42	43.30%	18	39.13%	

**Table 9:** The average values of MIC, MIC range, and MIC_50_ (mg/L) for selected antibiotics according to *S. mutans *biotypes set out in the arbitrary method.

	Biotypes from the arbitrary method			
	I	II	III	IV
All strains				
Amoxicillin				
MIC, range	0.007–0.5	0.007–0.5	0.007–0.5	0.007–0.25
MIC_50_	0.05	0.05	0.05	0.05
MIC, mean	0.092	0.059	0.084	0.042
Cefazolin				
MIC, range	0.02–1.5	0.02–1.5	0.02–1.5	0.03–1.5
MIC_50_	0.07	0.07	0.07	0.07
MIC, mean	0.153	0.152	0.158	0.169
Teicoplanin				
MIC, range	0.1–1.5	0.1–1.5	0.1–1.5	0.1–1
MIC_50_	0.5	0.5	0.5	0.5
MIC, mean	0.73	0.717	0.698	0.606
Erythromycin				
MIC, range	0.015–1	0.015–1	0.015–1	0.04–1
MIC_50_	0.07	0.07	0.07	0.07
MIC, mean	0.097	0.081	0.136	0.188
Noncavitated group				
Amoxicillin				
MIC, range	0.007–0.5	0.015–0.5	0.007–0.5	0.015–0.05
MIC_50_	0.05	0.05	0.05	0.05
MIC, mean	0.096	0.08	0.078	0.012
Cefazolin				
MIC, range	0.02–1.5	0.02–0.2	0.02–1.5	0.03–0.1
MIC_50_	0.07	0.07	0.07	0.07
MIC, mean	0.153	0.15	0.153	0.15
Teicoplanin				
MIC, range	0.1–1.5	0.1–1.5	0.1–1.5	0.1–1
MIC_50_	0.5	0.5	0.5	0.5
MIC, mean	0.734	0.744	0.726	0.7
Erythromycin				
MIC, range	0.015–1	0.015–0.5	0.015–1	0.04–0.07
MIC_50_	0.07	0.07	0.07	0.07
MIC, mean	0.097	0.061	0.091	0.05
Cavitated group				
Amoxicillin				
MIC, range	0.015–0.5	0.007–0.5	0.007–0.5	0.007–0.25
MIC_50_	0.05	0.05	0.04	0.04
MIC, mean	0.077	0.039	0.099	0.052
Cefazolin				
MIC, range	0.02–1.5	0.02–1.5	0.02–1.5	0.03–1.5
MIC_50_	0.07	0.07	0.08	0.08
MIC, mean	0.154	0.154	0.171	0.175
Teicoplanin				
MIC, range	0.1–1.5	0.1–1.5	0.1–1.5	0.1–1
MIC_50_	0.5	0.5	0.5	0.35
MIC, mean	0.716	0.692	0.627	0.575
Erythromycin				
MIC, range	0.015–1	0.015–1	0.015–1	0.04–1
MIC_50_	0.07	0.07	0.07	0.07
MIC, mean	0.098	0.099	0.252	0.233

**Table 10:** The average values of MIC, MIC range, and MIC_50_ (mg/L) for selected antibiotics according to *S. mutans *clusters/biotypes set out in the clusterization method.

	Clusters from the clusterization method		
	A	B	C
All strains			
Amoxicillin			
MIC, range	0.015–0.05	0.007–0.5	0.007–0.5
MIC_50_	0.05	0.05	0.05
MIC, mean	0.012	0.085	0.09
Cefazolin			
MIC, range	0.03–0.1	0.02–1.5	0.02–1.5
MIC_50_	0.07	0.07	0.07
MIC, mean	0.15	0.153	0.16
Teicoplanin			
MIC, range	0.1–1	0.1–.5	0.1–1.5
MIC_50_	0.5	0.5	0.5
MIC, mean	0.7	0.729	0.69
Erythromycin			
MIC, range	0.04–0.07	0.015–1	0.015–1
MIC_50_	0.07	0.07	0.07
MIC, mean	0.05	0.092	0.151
Noncavitated group			
Amoxicillin			
MIC, range	N/A	0.007–0.5	0.007–0.5
MIC_50_		0.05	0.05
MIC, mean		0.089	0.083
Cefazolin			
MIC, range		0.02–1.5	0.02–1.5
MIC_50_		0.07	0.07
MIC, mean		0.152	0.154
Teicoplanin			
MIC, range		0.1–1.5	0.1–1.5
MIC_50_		0.5	0.5
MIC, mean		0.737	0.724
Erythromycin			
MIC, range		0.015–1	0.015–1
MIC_50_		0.07	0.07
MIC, mean		0.082	0.1
Cavitated group			
Amoxicillin			
MIC, range	0.015–0.05	0.007–0.5	0.007–0.5
MIC_50_	0.05	0.05	0.04
MIC, mean	0.012	0.074	0.107
Cefazolin			
MIC, range	0.03–0.1	0.02–1.5	0.02–1.5
MIC_50_	0.07	0.07	0.08
MIC, mean	0.15	0.156	0.175
Teicoplanin			
MIC, range	0.1–1	0.1–1.5	0.1–1.5
MIC_50_	0.5	0.5	0.35
MIC, mean	0.7	0.699	0.611
Erythromycin			
MIC, range	0.04–0.07	0.015–1	0.015–1
MIC_50_	0.07	0.07	0.07
MIC, mean	0.05	0.126	0.271
